# Eye movement combined machine learning methods for three‐classification diagnosis of Alzheimer's disease

**DOI:** 10.1002/alz.087288

**Published:** 2025-01-09

**Authors:** Jiaqi Song, Weihua Yu, Yang Lü

**Affiliations:** ^1^ Chongqing medical university, Chongqing China; ^2^ Chongqing Medical University, Chongqing China; ^3^ The First Affiliated Hospital of Chongqing Medical University, Chongqing, Chongqing China

## Abstract

**Background:**

Alzheimer's disease (AD) is a chronically progressive neurodegenerative disease, and mild cognitive impairment (MCI) is a transitional stage between normal cognition and AD. Multi‐classification of AD poses a challenging task, and currently, only a few algorithms have achieved an accuracy above 60%. This study was designed to construct a diagnostic model based on eye movement parameters to distinguish dementia due to AD, MCI and normal cognition.

**Method:**

Eye movement data were collected from 258 subjects, comprising 66 individuals with normal cognition, 81 patients with MCI and 111 patients with dementia due to AD. Machine learning methods were used to construct classification models. Pearson's correlation analysis was used to detect correlations between the five most important eye movement indicators and neuropsychological scales.

**Result:**

Among all the machine learning classifiers, the gradient boosting classifier model demonstrated the best classification performance, achieved 68.2% of accuracy and 66.32% of F1‐score. Additionally, correlation analysis indicated that the eye movement parameters exhibited a significant correlation with various cognitive functions, including general cognitive status, attention, visuospatial ability, episodic memory, language and instrumental activities of daily life.

**Conclusion:**

Eye movement parameters in conjunction with machine learning methods achieve satisfactory overall accuracy, making it an effective and less time‐consuming method to assist clinical diagnosis of AD.

